# Urate Transporter 1 Can Be a Therapeutic Target Molecule for Chronic Kidney Disease and Diabetic Kidney Disease: A Retrospective Longitudinal Study

**DOI:** 10.3390/biomedicines11020567

**Published:** 2023-02-15

**Authors:** Hidekatsu Yanai, Hisayuki Katsuyama, Mariko Hakoshima, Hiroki Adachi

**Affiliations:** Department of Diabetes, Endocrinology and Metabolism, National Center for Global Health and Medicine Kohnodai Hospital, Chiba 272-8516, Japan

**Keywords:** chronic kidney disease, dotinurad, estimated glomerular filtration rate, serum creatinine, urate transporter 1, urinary uric acid excretion

## Abstract

Chronic kidney disease (CKD) is a major global health problem for which there are no curative drug treatments. Hyperuricemia is one of risk factors for CKD. The evidence on effects of uric acid (UA)-lowering treatments on the progression of CKD was very limited and previous meta-analyses used only trials which primarily used xanthin oxidase (XO) inhibitors because the reports on fulminant hepatitis due to benzbromarone kept us from using uricosuric agents for hyperuricemia patients. Dotinurad, a novel selective urate reabsorption inhibitor for the treatment of hyperuricemia, reduces serum UA levels by selectively inhibiting urate transporter 1 (URAT1). We retrospectively picked up patients who had taken dotinurad from June 2018 to August 2021 and compared metabolic parameters at baseline with the data at 3 and 6 months after the start of dotinurad. We found 84 patients, and approximately 74% of patients were complicated with CKD. After the start of dotinurad, improvements in serum lipids, systolic blood pressure, body weight, and albuminuria, in addition to reduction in serum UA, were observed. Dotinurad increased urinary UA excretion, and was effective to reduce serum UA in patients with both UA underexcretion type and renal UA overload type. Furthermore, urinary UA excretion was significantly and negatively correlated with serum creatine levels at baseline and at 6 months after the start of dotinurad, and the change in urinary UA excretion after 3 months was significantly and negatively correlated with change in serum creatine levels. The property of dotinurad, which selectively inhibits URAT1, but not other UA transporters, such as ATP-binding cassette, subfamily G, and 2 (ABCG2), which ABCG2 is a UA and uremic toxin exporter, may be beneficially associated with pathology of CKD. URAT1 can be a therapeutic target molecule for CKD and DKD.

## 1. Introduction

Chronic kidney disease (CKD) is a major global health problem for which there are no curative drug treatments. Lifestyle-related diseases, such as obesity, hypertension, diabetes, and dyslipidemia, are currently important risk factors for CKD [1]. Evidence supports a pathophysiological role for overactivation of the mineralocorticoid receptor in cardiorenal diseases, including CKD and diabetes, through inflammation and fibrosis that lead to progressive kidney and cardiovascular dysfunction [2]. A nonsteroidal, selective mineralocorticoid receptor antagonist (MRA), finerenone, had more potent anti-inflammatory and anti-fibrotic effects than steroidal mineralocorticoid receptor antagonists in preclinical models [3]. Very recently, the treatment with a selective MRA resulted in lower risks of CKD progression than placebo in patients with CKD and type 2 diabetes [4]. The treatments for diabetes and obesity, such as sodium-glucose co-transporter 2 (SGLT2) inhibitors and glucagon-like peptide-1 receptor agonists (GLP-1RA), are emerging as promising treatment options in CKD and diabetic kidney disease (DKD) [5,6,7,8,9,10,11]. The therapeutic intervention for risk factors, such as hypertension, obesity, and diabetes, have shown to reduce the onset and progression of CKD and DKA, and also have elucidated the molecular mechanisms for the progression of CKD and DKD.

Hyperuricemia is also a lifestyle-related disease, but most patients with hyperuricemia have no symptoms. Some studies recommend lower serum uric acid (UA) values < 5 mg/dL for the apparently healthy population, while the European League Against Rheumatism recommends a target of <6.0 mg/dL with medication for anti-hyperuricemia [12,13,14]. Can the therapeutic intervention for hyperuricemia, the remaining risk factor for CKD, affect the progression of CKD? In the meta-analysis, the relative risk (RR) of CKD was 1.22 (95% confidence interval [CI], 1.16 to 1.28) per 1 mg/dL serum UA level increment [15]. Another meta-analysis showed a significant positive association between elevated serum UA levels and the new-onset CKD at follow-ups (odds ratio [OR], 1.15; 95% CI, 1.05 to 1.25) [16]. Hyperuricemia was found be an independent predictor for the development of newly diagnosed CKD. The meta-analyses which studied whether UA-lowering treatment (ULT) reduces the progression of CKD showed insufficient data on incidence of end-stage renal disease (ESRD) for analysis and the heterogeneity across included studies, suggesting that adequately powered randomized controlled trials (RCTs) are needed to establish whether ULT has beneficial renal effects [17,18]. The RCTs included in these meta-analyses on the effect of UTL on the onset and progression of CKD primarily used xanthin oxidase (XO) inhibitors, such as allopurinol and febuxostat.

A case–control study of 100 patients with primary gout and 72 healthy controls was undertaken [19]. Most patients with gout showed lower UA clearance, fractional excretion of UA, excretion of UA per volume of glomerular filtration, and urinary UA to creatinine ratio than controls, suggesting that renal underexcretion is the main mechanism for the development of primary hyperuricemia. Therefore, considering the pathology of hyperuricemia, it was more reasonable to use uricosuric agents, such as benzbromarone, than XO inhibitors for the treatment of hyperuricemia. However, the reports on fulminant or sub-fulminant hepatitis due to benzbromarone kept us from using uricosuric agents for hyperuricemia patients [20,21].

Dotinurad, a novel selective urate reabsorption inhibitor (SURI) that has a potential for the treatment of hyperuricemia, reduces serum UA levels by selectively inhibiting urate transporter 1 (URAT1) [22]. The RCT to evaluate the efficacy and safety of dotinurad in hyperuricemic Japanese patients (*n* = 80) with or without gout was performed [23], and dotinurad has been shown to have a substantial serum UA lowering effect in patients with hyperuricemia without serious adverse event. Very recently, we experienced a type 2 diabetic patients with CKD stage G4 whose serum creatinine and estimated glomerular filtration rate (eGFR) were remarkably improved by the additional use of dotinurad to SGLT2 inhibitor, GLP-1RA, angiotensin receptor blocker (ARB), and MRA, finerenone [24].

Here, we aimed to study the Influences of dotinurad on metabolic parameters, including the markers for renal function and UA excretion in patients with hyperuricemia.

## 2. Materials and Methods

### 2.1. Study Population

The study protocol was approved by the Ethics Committee of the National Center for Global Health and Medicine (NCGM-S-004615-00), and the study was performed in accordance with the Declaration of Helsinki.

We retrospectively picked patients who had taken dotinurad for the treatment of asymptomatic hyperuricemia, from September 2021 to December 2022, and compared metabolic parameters at baseline with the data at 3 and 6 months after the start of dotinurad. Information about medical history and medication were obtained via an electronic medical record. Such information included age, gender, body weight, body mass index (BMI), systolic and diastolic blood pressures, comorbidities, and treatments for type 2 diabetes, hypertension, dyslipidemia, and hyperuricemia. Informed consent was obtained by the opt-out approach. According to the diagnostic criteria by the Japan Diabetes Society, the Japanese Society of Hypertension, Japan Atherosclerosis Society; Japanese Society of Gout and Uric and Nucleic Acids, we defined type 2 diabetes as taking anti-diabetic drugs and/or HbA1c > 6.5%; hypertension as taking anti-hypertensive drugs and/or systolic blood pressure ≥140 mmHg and/or diastolic blood pressure ≥90 mmHg; dyslipidemia as taking anti-hyperlipidemic drugs and/or low-density lipoprotein-cholesterol (LDL-C) ≥ 140 mg/dL and/or triglyceride (TG) ≥ 150 mg/dL and/or triglyceride high-density lipoprotein-cholesterol (HDL-C) < 40 mg/dL; hyperuricemia as taking uric acid (UA) lowering drugs and/or serum UA ≥ 7.0 mg/dL, respectively. According to the diagnostic criteria by the Japanese Society of Nephrology, CKD was defined as reduced eGFR (eGFR < 60 mL/min/1.73 m^2^) or proteinuria including albuminuria (urinary albumin/creatinine [UACR] ≥ 30).

### 2.2. Laboratory Measurements

Serum alanine aminotransferase (ALT), aspartate aminotransferase (AST), and gamma-glutamyl transferase (GGT) were measured by a modified Japan Society of Clinical Chemistry (JSCC) reference method. Serum albumin was measured by the modified bromocresol purple (BCP) method. Serum UA was measured by uricase peroxidase method. Plasma glucose was the hexokinase UV method. Hemoglobin A1c (HbA1c) was measured by automated enzyme-linked immunosorbent assays (TOSOH, Tokyo, Japan). Serum creatinine, TG, HDL-C, and LDL-C were determined enzymatically. The eGFR was calculated by using the Chronic Kidney Disease Epidemiology Collaboration (CKD–EPI) formula. To understand urinary excretion of UA, we measured urinary UA/creatinine.

### 2.3. Phenotyping of Hyperuricemia

Hyperuricemia has been previously classified into the “UA overproduction type”, “UA underexcretion type”, and “combined type” [25]. ATP-binding cassette, subfamily G, 2 (ABCG2)-knockout mice showed increased serum UA and renal UA excretion, and decreased intestinal UA excretion [26], indicating that a significance of decreased extra-renal UA excretion caused by ABCG2 dysfunction for hyperuricemia. At present, hyperuricemia is classified into “renal UA overload type” (“UA extra-renal underexcretion type” and “UA overproduction type”), “UA underexcretion type”, and “combined type” [25]. Yamanaka et al. previously suggested that “UA overproduction type” and “UA underexcretion type” showed urinary UA/creatinine >0.5 and ≤0.5, respectively [27]. Therefore, we defined patients with urinary UA/creatinine >0.5 and ≤0.5 at baseline as “renal UA overload type” and “UA underexcretion type”, respectively.

### 2.4. Adjustment of Concurrent Treatments for Lifestyle-Related Diseases except for Hyperuricemia

Anti-hypertensive drugs, such as angiotensin receptor blockers (ARB), and anti-hyperlipidemic drugs, such as statin and SGLT2 inhibitors [9], may affect metabolic parameters, such as blood pressure, serum lipids, and body weight. Therefore, we analyzed the effects of dotinurad on metabolic parameters by dividing into patients with and without anti-hypertensive drugs (ARB and calcium channel blockers), and by dividing into patients with and without anti-hyperlipidemic drugs (statin, pemafibrate, and ezetimibe), and by dividing into patients with and without SGLT2 inhibitors. Furthermore, we studied the effect of dotinurad in patients who had not taken anti-hypertensive and anti-hyperlipidemic drugs and SGLT2 inhibitors.

### 2.5. Statistical Analysis

Statistical analyses were performed by using SPSS version 23 (IBM Co., Ltd., Chicago, IL, USA). All values are expressed as the mean  ±  standard deviation, except for sex. The paired t-test was used to statistically analyze comparison in metabolic parameters between before and after the start of dotinurad. Correlations between changes in two parameters were statistically analyzed by the Spearman’s correlation (non-parametric). *p* value of <0.05 and <0.1 was considered to be statistically significant, and to have tendency, respectively.

## 3. Results

### 3.1. Baseline Characteristics of Patients Studied

We found 84 patients, and baseline characteristics for patients who had taken dotinurad were shown in Table 1. Dotinurad was prescribed to patients with asymptomatic hyperuricemia. The mean BMI was over 27 kg/m^2^, indicating that our study included a relatively large number of overweight patients. Almost 60% of patients had type 2 diabetes, hypertension, and dyslipidemia. Very high-percentage (approximately 74%) of patients were complicated with CKD.

Thirty-five patients had not used UA-lowering drugs, and forty-nine patients had undergone the switching from XO inhibitors, such as febuxostat, topiroxostat, and allopurinol, to dotinurad. A half of patients had taken daily 0.5 mg of dotinurad, and 44% of patients had taken daily 1.0 mg of dotinurad.

### 3.2. Concurrent Treatments for Hypertension, Dyslipidemia and Type 2 Diabetes of Patients Studied

Treatments for hypertension, dyslipidemia, and type 2 diabetes at baseline were shown in Table 2. Forty-five percentage of patients had taken ARB and statins, and one-third of patients had taken SGLT2 inhibitors. Such treatments did not change during the study period.

### 3.3. Changes in Metabolic Parameters after the Start of Dotinurad

#### 3.3.1. Changes in Metabolic Parameters after the Start of Dotinurad in All Patients

Changes in metabolic parameters after the start of dotinurad were shown in Table 3. Body weight, blood pressures, transaminase, glucose, and lipid metabolism did not show any changes. Serum UA did not show a significant change at 3 months after the start of dotinurad as compared with baseline; however, serum UA significantly decreased after 6 months. Interestingly, UACR tended to decrease at 6 months after the start of dotinurad.

#### 3.3.2. Changes in Metabolic Parameters after the Start of Dotinurad in Patients Who Had Not Used UA-Lowering Drugs

Changes in metabolic parameters after the start of dotinurad in patients who had not used UA-lowering drugs were shown in Table 4.

Body weight, blood pressures, transaminases, and glucose metabolism did not show any changes. Serum LDL-C significantly decreased and non-HDL-C tended to decrease at 3 months after the start of dotinurad as compared with baseline. Serum UA significantly decreased at both 3 and 6 months after the start of dotinurad. UACR tended to decrease at 6 months after the start of dotinurad.

#### 3.3.3. Changes in Metabolic Parameters after the Start of Dotinurad in Patients Who Switched from XO Inhibitors

Changes in metabolic parameters after the start of dotinurad in in patients who switched from XO inhibitors were shown in Table 5.

Body weight tended to decrease at 6 months after the start of dotinurad as compared with baseline. Systolic blood pressure significantly decreased at 3 months after the start of dotinurad as compared with baseline. Transaminases, glucose, and lipid metabolism, eGFR and UACR did not show any changes. Serum UA significantly increased at 3 months after the start of dotinurad as compared with baseline; however, serum UA did not show a significant difference at 6 months after the start of dotinurad.

### 3.4. Changes in Urinary UA/Creatinine after the Start of Dotinurad

**Figure 1:**
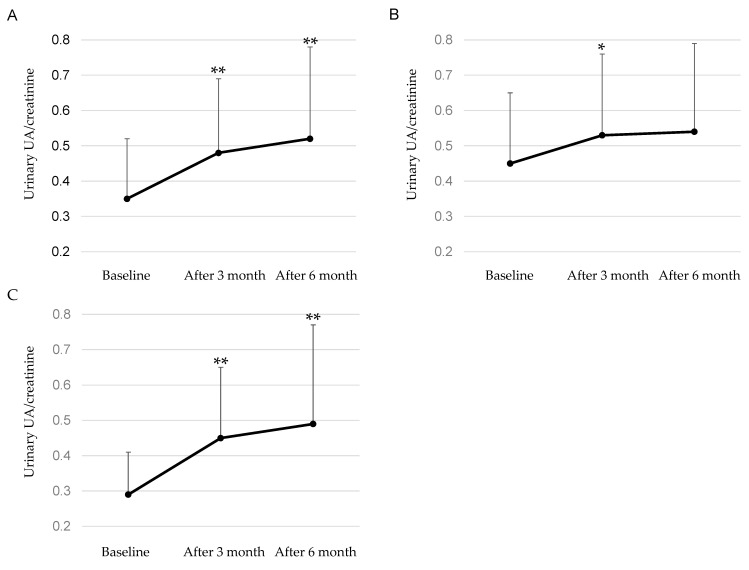
Changes in urinary UA/creatine after the start of dotinurad in all patients (**A**), patients who had not used UA-lowering drugs (**B**), and patients who switched from XO inhibitors (**C**). *, ** indicate *p* < 0.1 and *p* < 0.05, respectively.

Changes in urinary UA/creatine after the start of dotinurad in patients were shown in Figure 1. Urinary UA/creatine significantly increased at both 3 and 6 months after the start of dotinurad as compared with baseline in all patients. Among patients who had not used UA-lowering drugs, urinary UA/creatine tended to increase at 3 months after the start of dotinurad as compared with baseline, however, urinary UA/creatine did not show a significant change after 6 months. In patients who switched from XO inhibitors, urinary UA/creatine significantly increased at both 3 and 6 months after the start of dotinurad as compared with baseline.

### 3.5. Effects of Concurrent Treatments for Hypertension, Dyslipidemia and Type 2 Diabetes on Metabolic Parameters

#### 3.5.1. Differences in Effects of Dotinurad on Metabolic Parameters between Patients with and without Anti-Hypertensive Drugs

Changes in metabolic parameters after the start of dotinurad in patients who had taken anti-hypertensive drugs (ARB and calcium channel blockers) were shown in Table 6. Urinary UA/creatinine significantly increased at 3 and 6 months after the start of dotinurad. Serum UA tended to decrease after 3 months and significantly decreased after 6 months. UACR tended to decrease after 6 months.

Changes in metabolic parameters after the start of dotinurad in patients who had not taken anti-hypertensive drugs were shown in Table 7. Urinary UA/creatinine showed a significant increase at both 3 and 6 months after the start of dotinurad. However, serum UA did not show a significant change. Body weight and systolic blood pressure tended to decrease at both 3 and 6 months after the start of dotinurad. Serum TG tended to decrease after 3 months. Serum creatine significantly increased and eGFR significantly decreased after 3 months.

#### 3.5.2. Differences in Effects of Dotinurad on Metabolic Parameters between Patients with and without Anti-Hyperlipidemic Drugs

Changes in metabolic parameters after the start of dotinurad in patients who had taken anti-hyperlipidemic drugs (statin, pemafibrate, and ezetimibe) were shown in Table 8. Urinary UA/creatinine significantly increased at both 3 and 6 months after the start of dotinurad. Serum UA did not show a significant change. Diastolic blood pressure tended to decrease after 3 months. UACR tended to decrease after 6 months.

Changes in metabolic parameters after the start of dotinurad in patients who had not taken anti-hyperlipidemic drugs were shown in Table 9. Urinary UA/creatinine showed a significant increase at both 3 and 6 months after the start of dotinurad. Serum UA tended to decrease after 6 months. Body weight tended to decrease at 3 months after the start of dotinurad. Interestingly, HbA1c tended to decrease after 6 months.

#### 3.5.3. Differences in Effects of Dotinurad on Metabolic Parameters between Patients with and without SGLT2 Inhibitors

Changes in metabolic parameters after the start of dotinurad in patients who had also taken SGLT2 inhibitors were shown in Table 10. Only urinary UA/creatinine showed a significant increase at 3 months after the start of dotinurad. Serum UA did not show a significant difference. Diastolic blood pressure tended to increase after 6 months.

Changes in metabolic parameters after the start of dotinurad in patients who had not taken SGLT2 inhibitors were shown in Table 11. Urinary UA/creatinine showed a significant increase at both 3 and 6 months after the start of dotinurad. Serum UA also showed a significant decrease at both 3 and 6 months after the start of dotinurad. Very interestingly, HbA1c significantly decreased at 6 months after the start of dotinurad.

#### 3.5.4. Changes in Metabolic Parameters after the Start of Dotinurad in Patients Who Had Not Taken Anti-Hypertensive and Anti-Hyperlipidemic Drugs and SGLT2 Inhibitors

Changes in metabolic parameters after the start of dotinurad in patients who had not taken anti-hypertensive and anti-hyperlipidemic drugs and SGLT2 inhibitors were shown in Table 12. Urinary UA/creatinine showed a significant increase at both 3 and 6 months after the start of dotinurad. Serum UA did not show a significant change. Body weight tended to decrease at 3 months after the start of dotinurad. HbA1c tended to decrease after 6 months.

### 3.6. Phenotypes of Hyperuricemia and Effect of Dotinurad on Urinary UA/Creatinine

**Figure 2:**
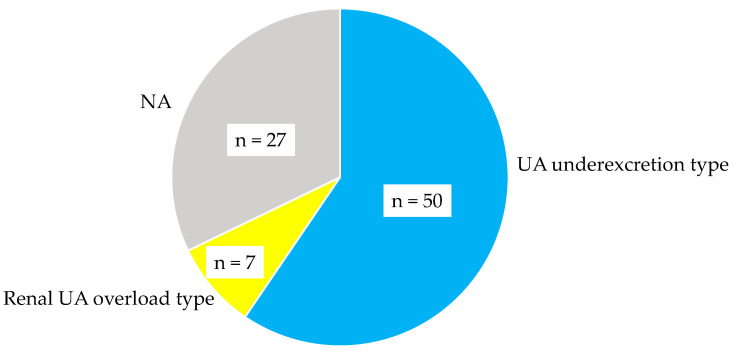
The proportion of hyperuricemia phenotype in our patients. NA, not available.

The proportion of hyperuricemia phenotype in our patients were shown in Figure 2. Among patients whose urinary UA/creatinine were measured, almost 88% patients were UA underexcretion type, and the proportion of patients with renal UA overload type was only 12%.

**Figure 3:**
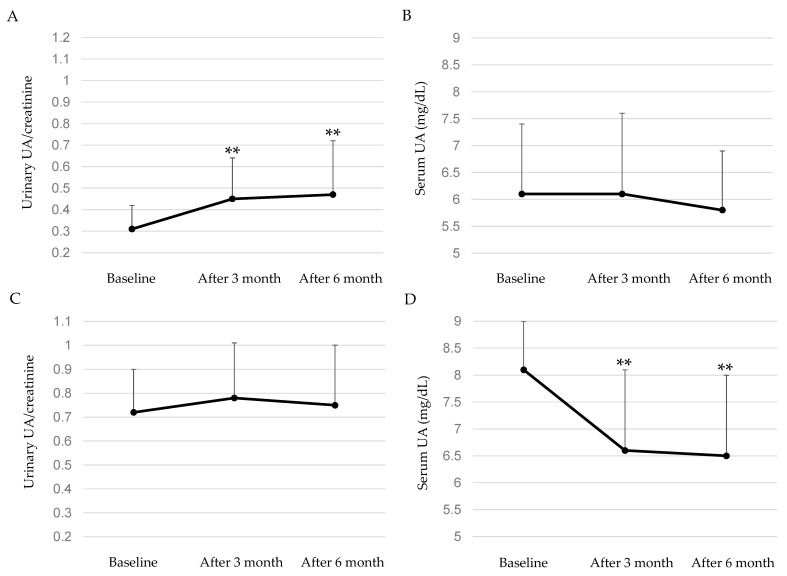
The effect of dotinurad on urinary UA/creatinine and serum UA levels in patients with UA underexcretion type (**A**,**B**), and in patients with renal UA overload type (**C**,**D**). ** *p* < 0.05.

The effect of dotinurad on urinary UA/creatinine and serum UA levels in each hyperuricemia phenotype were shown in Figure 3. In patients with UA underexcretion type, dotinurad significantly increased urinary UA/creatinine at both 3 and 6 months after the start of dotinurad as compared with baseline. However, there were no significant changes in serum UA after the start of dotinurad. In patients with renal UA overload type, there were no significant changes in urinary UA/creatinine after the start of dotinurad. However, dotinurad significantly reduced serum UA at both 3 and 6 months after the start of dotinurad as compared with baseline.

### 3.7. The Association of Urinary UA/Creatinie with Serum Creatinie and eGFR

#### 3.7.1. Correlations between Urinary UA/Creatinine and Serum Creatinine

**Figure 4:**
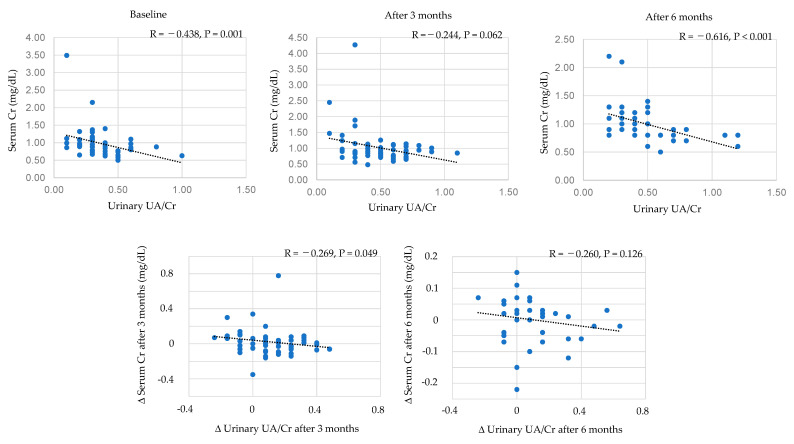
Correlations between urinary UA/creatinine and serum creatinine. Cr, creatinine.

Correlations between urinary UA/creatinine and serum creatinine were shown in Figure 4. Urinary UA/creatine was significantly and negatively correlated with serum creatine levels at baseline and at 6 months after the start of dotinurad, and urinary UA/creatine tended to be negatively correlated with serum creatine levels after 3 months. The change in urinary UA/creatine after 3 months was significantly and negatively correlated with change in serum creatine.

#### 3.7.2. Correlations between Urinary UA/Creatinine and eGFR

**Figure 5:**
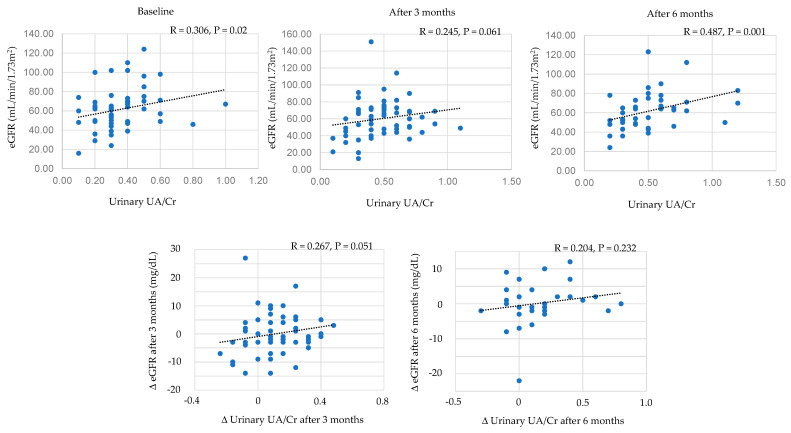
Correlations between urinary UA/creatinine and eGFR. Cr, creatinine.

Correlations between urinary UA/creatinine and eGFR were shown in Figure 5. Urinary UA/creatine was significantly and positively correlated with eGFR at baseline and at 6 months after the start of dotinurad, and urinary UA/creatine tended to be positively correlated with eGFR after 3 months. Change in urinary UA/creatine tended to be positively correlated with change in eGFR after 3 months.

### 3.8. The Association of Serum UA with Serum Creatinie and eGFR

#### 3.8.1. Correlations between Serum UA and Serum Creatinine

**Figure 6:**
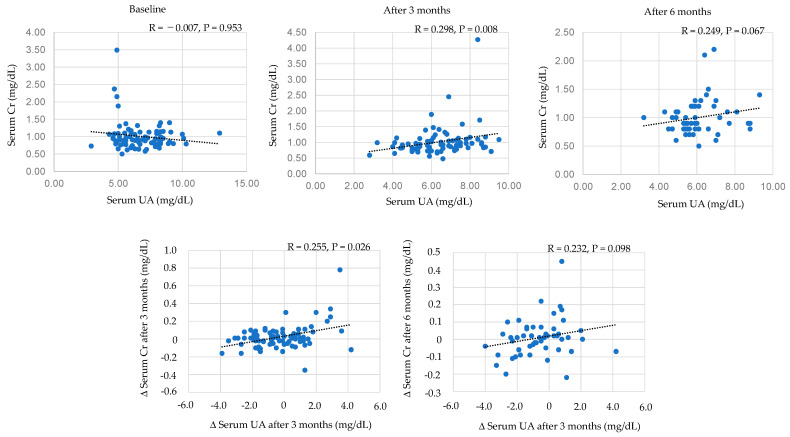
Correlations between serum UA and serum creatinine. Cr, creatinine.

Correlations between serum UA and serum creatinine were shown in Figure 6. Serum UA level was significantly and positively correlated with serum creatine levels at 3 months after the start of dotinurad, and serum UA level tended to be positively correlated with serum creatine level after 6 months. Change in serum UA was significantly and positively correlated with change in serum creatine levels at 3 months after the start of dotinurad, and the change in serum UA tended to be positively correlated with change in serum creatine levels at 6 months after the start of dotinurad.

#### 3.8.2. Correlations between Serum UA and eGFR

**Figure 7:**
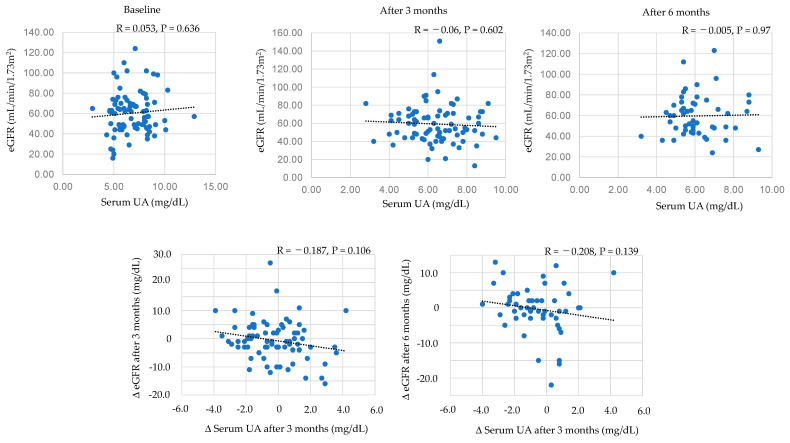
Correlations between serum UA and eGFR.

Correlations between serum UA and eGFR were shown in Figure 7. Serum UA levels were not correlated with eGFR at any time after the start of dotinurad. Furthermore, change in serum UA levels were not correlated with change in eGFR at both 3 and 6 months after the start of dotinurad.

## 4. Discussion

Almost 60% of our patients had type 2 diabetes, and over 70% of patients have CKD; therefore, present study could observe the effect of a novel selective URAT1 inhibitor, dotinurad on metabolic and renal parameters in CKD and DKD patients. In other words, this study may clarify a clinical significance of URAT1 in CKD and DKD. Renal excretion of UA is the major regulator of serum UA concentration [28,29,30]. In humans, reabsorption of UA into the blood plays a crucial role to regulate serum UA. The UA exchange is mediated by various molecules, such as URAT1, glucose transporter 9 (GLUT9), and ABCG2, expressed in renal proximal tubule [31,32,33,34]. Renal UA reabsorption is mainly mediated by URAT1 and GLUT9 [33,35,36]. URAT1 is found in the apical membrane of proximal tubule epithelial cells [37]. In addition, ABCG2 has been identified as a high-capacity UA exporter that mediates renal and/or extra-renal UA excretion [38]. ABCG2 is now known to be involved as well in UA excretion into the intestine [38].

We found that dotinurad improved UACR in addition to serum UA in all patients and patients who had not used UA-lowering drugs. An increase in urinary UA excretion measured by urinary UA/creatine can indicate the inhibitory effect of URAT1 by dotinurad. Dotinurad significantly increased urinary UA/creatine in all patients, especially in patients who switched from XO inhibitors. As almost 88% of our patients were UA underexcretion type, such patients should have been treated by uricosuric agents. As the effect of dotinurad on urinary UA/creatinine in each hyperuricemia phenotype showed, dotinurad significantly increased urinary UA/creatinine in patients with UA underexcretion type. More interesting is the effect of dotinurad on urinary UA/creatinine and serum UA levels in patients with renal UA overload type. In patients with renal UA overload type, there were no significant changes in urinary UA/creatinine by dotinurad. However, dotinurad remarkably reduced serum UA as compared with baseline. On the other hand, in patients with UA underexcretion type, despite significant increase in urinary UA/creatine, the decrease in serum UA levels was modest. This suggests that expression and/or function of URAT1 in patients with UA underexcretion type may be higher than those in patients with renal UA overload type. In short, while the same dose of dotinurad increases urinary UA excretion, the effect on serum UA levels may be insufficient in patients with UA underexcretion type with higher expressed URAT1; the same dose of dotinurad increases urinary UA excretion, but the effect on serum UA levels may be greater in patients with renal UA overload type with lower expressed URAT1. The increased protein level of URAT1 was observed in obesity/metabolic syndrome model mice [39]. Upon high-purine load, insulin resistance enhances UA reabsorption as manifested by up-regulated URAT1 expression and reduces UA excretion in the Otsuka-Long-Evans-Tokushima Fatty rats [40]. UA underexcretion type may be associated with insulin resistance, which is strongly involved in the development of CKD and DKD. Furthermore, this result suggests that dotinurad is effective to reduce serum UA by using appropriate dose in patients with both UA underexcretion type and renal UA overload type.

We analyzed the correlation of urinary UA/creatinine with the markers for CKD/DKD, such as serum creatinine levels and eGFR. Urinary UA/creatinine was strongly associated with lower serum creatinine levels and higher eGFR, and the increase in urinary UA/creatinine by dotinurad was also associated with an improvement of serum creatinine levels and eGFR. However, the influence of serum UA on serum creatinine levels was weak, and serum UA levels were not associated with eGFR. This suggests that urinary UA excretion may perform a more crucial role in progression of CKD/DKA as compared with serum UA levels.

**Figure 8:**
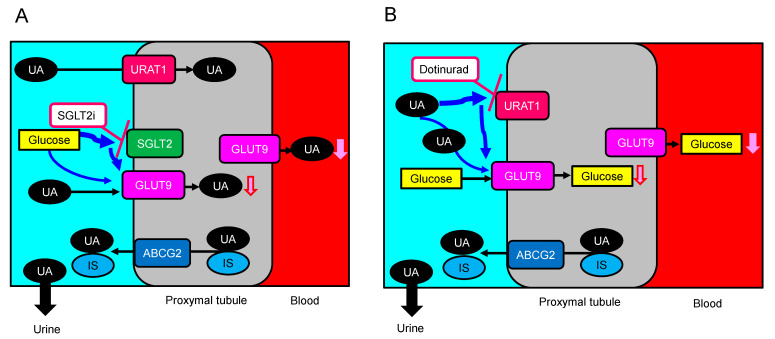
Crosstalk among UA transporters and glucose transporters, induced by SGLT2i (**A**) and dotinurad (**B**). ABCG2, ATP-binding cassette, subfamily G; GLUT9, glucose transporter 9; IS, indoxyl sulfate; SGLT2i, sodium-glucose cotransporter 2 inhibitors; UA, uric acid; URAT1, urate transporter 1.

We found an improvement in serum lipids, such as reduction in LDL-C and non-HDL-C by dotinurad, in patients who had not used UA-lowering drugs. The meta-analysis showed that hyperuricemia increased the likelihood of dyslipidemia, and the pooled OR for the highest UA level vs. the lowest UA level was 1.84 (95%CI, 1.49 to 2.28) [41]. A reduction in serum UA by dotinurad may improve serum lipids. We found an improvement in systolic blood pressure by dotinurad in patients who switched from XO inhibitors. The meta-analysis showed that hyperuricemia was associated with a higher risk of incident hypertension [42]. Adjusted RR was 1.15 (95%CI, 1.06 to 1.26) for a 1 mg/dL increase in serum UA. Another meta-analysis showed that hyperuricemia was associated with an increased risk for incident hypertension (adjusted RR, 1.41; 95%CI, 1.23 to 1.58) [43]. For a 1 mg/dL increase in UA level, the pooled RR for incident hypertension after adjusting for potential confounding was 1.13 (95%CI, 1.06 to 1.20). Hyperuricemia may be also responsible for microvascular damage through stimulation of the renin-angiotensin system (RAS), inhibition of endothelial nitric oxide, and proliferative effects on vascular smooth muscle cells [44]. Did the reduction in serum UA by dotinurad improve systolic blood pressure in our patients who switched from XO inhibitors? The answer is “No”. Serum UA significantly increased in patients who switched from XO inhibitors. Why was systolic blood pressure reduced by dotinurad? We also observed a reduction in body weight by dotinurad in patients who switched from XO inhibitors. As one of the causes that SGLT2 inhibitors suppress the progression of CKD, lowering of serum UA by SGLT2 has been suggested [9]. SGLT2 inhibitors increase the concentration of glucose in the proximal tubules, and glucose may compete with UA for apical GLUT9, reducing UA reabsorption [28,45] (Figure 8A). Dotinurad selectively inhibits URAT1 and increases the concentration of UA in the proximal tubules, and UA may compete with glucose for apical GLUT9, reducing glucose reabsorption (Figure 8B), which may induce an improvement of serum lipids, blood pressure, body weight, and UACR-like SGLT2 inhibitors [9]. In present study, HbA1c significantly decreased in patients who had not taken SGLT2 inhibitors at 6 months after the start of dotinurad, supporting our hypothesis. SGL2 inhibitors improve body weight, dyslipidemia, blood pressure, and UACR [9]. An improvement in body weight, dyslipidemia, blood pressure, and UACR after the start of dotinurad might have been induced by reduced glucose reabsorption by GLUT9.

**Figure 9:**
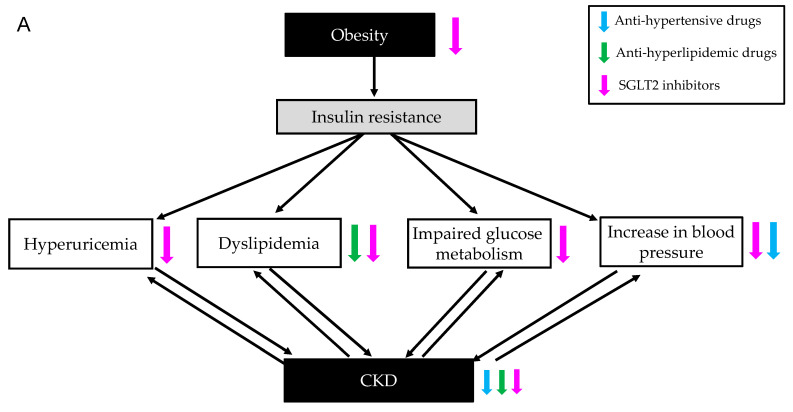

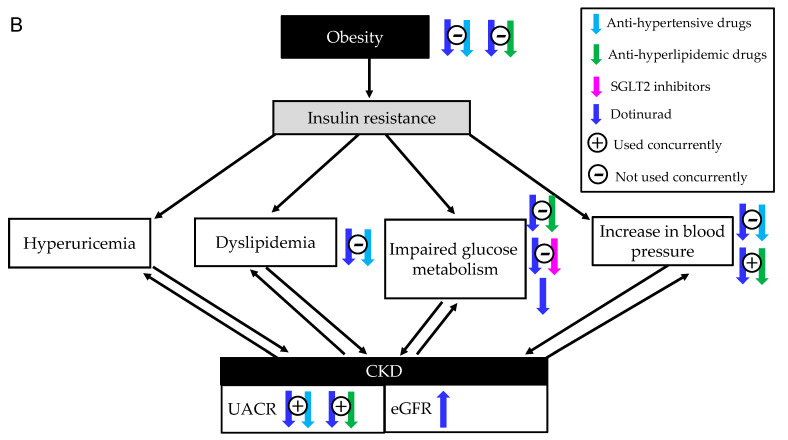
Relation of metabolic syndrome components and hyperuricemia with CKD, and effects of anti-hypertensive and anti-hyperlipidemic drugs and SGLT2 inhibitors on metabolic parameters and CKD (**A**). Effects of dotinurad with and without anti-hypertensive and anti-hyperlipidemic drugs and SGLT2 inhibitors on metabolic parameters and CKD (**B**).

We adjusted confounding variables, such as concurrent medications, for other lifestyle-related diseases except for hyperuricemia because such treatments have an effect on CKD outcome and metabolic parameters. Hyperuricemia was strongly associated with metabolic syndrome [28] and metabolic syndrome, and each factor of metabolic syndrome induce the development and progression of CKD (Figure 9A). CKD also induces the development and progression of hyperuricemia, dyslipidemia, impaired glucose metabolism, and hypertension [28,46].

Anti-hypertensive drugs, such as ARB and calcium channel blockers, have a favorable impact on blood pressure and renal parameters for CKD [47,48] (Figure 9A). Therefore, we analyzed the effects of dotinurad on metabolic parameters by dividing into patients with and without anti-hypertensive drugs. UACR tended to decrease in patients with anti-hypertensive drugs, suggesting a synergetic effect of dotinurad and anti-hypertensive drugs on UACR. Systolic blood pressure tended to decrease in patients without anti-hypertensive drugs, suggesting that dotinurad reduced blood pressure by possibly a relative inhibition of GLUT9, which was supported by reduction in body weight and serum TG in this population. In this population, serum creatine significantly increased and eGFR significantly decreased after 3 months, which may be due to reduced glomerular hyperfiltration because they lost body weight.

Anti-hyperlipidemic drugs, such as statins, fibrates, and ezetimibe, have beneficial effects on dyslipidemia and CKD [49,50,51,52] (Figure 9A). Diastolic blood pressure and UACR tended to decrease in patients with anti-hyperlipidemic drugs, suggesting a synergetic effect of dotinurad and anti-hypertensive drugs on blood pressure and UACR. Body weight tended to decrease and HbA1c significantly decreased in patients without anti-hyperlipidemic drugs, which may be also due to a relative inhibition of GLUT9.

SGLT2 inhibitors have beneficial effects on hyperuricemia, dyslipidemia, glucose metabolism, blood pressure, and CKD [9] (Figure 9A). Diastolic blood pressure tended to decrease in patients with SGLT2 inhibitors. Interestingly, HbA1c significantly decreased in patients without SGLT2 inhibitors, which may be also due to a relative inhibition of GLUT9.

Body weight, HbA1c tended to decrease and eGFR tended to increase in patients who had not taken anti-hypertensive and anti-hyperlipidemic drugs, and SGLT2 inhibitors, supporting a significance of a relative inhibition of GLUT-9 by dotinurad for an improvement of metabolic and renal parameters.

The summery of effects of dotinurad with and without concurrent medications for other lifestyle-related diseases except for hyperuricemia were shown in Figure 9B. An improvement of UACR seems to be due to a synergetic effect of dotinurad and anti-hypertensive, and anti-hyperlipidemic drugs. An improvement of body weight, dyslipidemia, glucose metabolism, and eGFR may be induced by a relative inhibition of GLUT9 or other mechanism, such as an increase in urinary UA excretion by dotinurad. However, such suggestion may be still premature because CKD is multifactorial disease and multi-disciplinary treatments are required for suppression of CKD. Further studies, preferably with a great number of patients, should be performed in the future.

ABCG2 is a high-capacity UA exporter, the dysfunction of which raises hyperuricemia risk [26] (Figure 8). Dotinurad had no effect on ABCG2 [22]. However, other UA-lowering drugs, such as benzbromarone (uricosuric agent) and febuxostat (XO inhibitor), have been reported to inhibit ABCG2 completely [53]. Topiroxostat (XO inhibitor) also inhibited ABCG2 by over 80% [53]. CKD patients accumulate uremic toxins in the body. ABCG2 was a major transporter of the uremic toxin, indoxyl sulfate (IS) [54] (Figure 8). ABCG2 regulates the pathophysiological excretion of IS and strongly affects CKD survival rates [55]. High selectivity of dotinurad for URAT1 may be beneficial for suppression of progression of CKD.

Limitations of the study need to be addressed. This is a cross-sectional study, limiting inferences of causality and its direction. Although we did not change treatments for diabetes and hypertension, dyslipidemia intentionally during the study period, we cannot deny the beneficial role of the concomitant assumption of other drugs including aspect of synergism and/or the possible interaction between dotinurad and other treatments for metabolic parameters. We classified hyperuricemic patients into “UA overproduction type” and “UA underexcretion type” based on urinary UA/creatinine ≥ or <0.5, which may be weak. We should have measured 24 h urine UA, and should have analyzed urate transporter variants. To elucidate this in further studies, preferentially, RCT, that including a large number of patients, should be performed in the future.

## 5. Conclusions

Dotinurad, a selective URAT1 inhibitor, improved serum lipids, blood pressure, body weight, and albuminuria, in addition to reduction in serum UA, in CKD/DKA patients. Present study suggested that dotinurad was effective to reduce serum UA by using appropriate dose in patients with both UA underexcretion type and renal UA overload type. Furthermore, present study suggested that an increase in urinary UA excretion was favorably associated with renal function. The property of dotinurad, which selectively inhibits URAT1, but not ABCG, may be beneficially associated with pathology of CKD. URAT1 can be a therapeutic target molecule for CKD and DKD.

## Figures and Tables

**Table 1:** Baseline characteristics for patients who had taken dotinurad (*n* = 84).

Clinical characteristics	
Age (years old)	64 ± 16
Gender (male/female)	55/31
Body weight (mean ± SD, kg)	76.7 ± 17.6
Body mass index (mean ± SD, kg/m^2^)	27.4 ± 8.4
Systolic blood pressure (mean ± SD, mmHg)	132.9 ± 18.7
Diastolic blood pressure (mean ± SD, mmHg)	76.3 ± 13.7
Comorbidities	
Dyslipidemia (*n*, %)	48, 55.8%
Hypertension (*n*, %)	55, 64.0%
Type 2 diabetes (*n*, %)	51, 59.3%
Chronic kidney disease (*n*, %)	62, 73.8%
Treatments for hyperuricemia	
Febuxostat (*n*, %)	38, 44.2%
10 mg/day (*n*)	29
20 mg/day (*n*)	7
40 mg/day (*n*)	2
Topiroxostat (*n*, %)	4, 4.7%
20 mg/day (*n*)	2
40 mg/day (*n*)	2
Allopurinol (*n*, %)	7, 8.1%
100 mg/day (*n*)	3
150 mg/day (*n*)	1
200 mg/day (*n*)	3
Daily dose of dotinurad	
0.5 mg/day (*n*, %)	42, 50.0%
1.0 mg/day (*n*, %)	37, 44.0%
2.0 mg/day (*n*, %)	5, 6.0%

**Table 2:** Treatments for hypertension, dyslipidemia and type 2 diabetes.

Treatments for hypertension	
Angiotensin receptor blockers (*n*, %)	39, 45.3%
Calcium channel blockers (*n*, %)	35, 41.7%
Treatments for dyslipidemia	
Statins (*n*, %)	39, 45.3%
Pemafibrate (*n*, %)	12, 14.3%
Ezetimibe (*n*, %)	12, 14.3%
Treatments for type 2 diabetes	
Sodium-glucose co-transporter 2 inhibitors (*n*, %)	25, 29.1%
Dipeptidyl peptidase inhibitors (*n*, %)	29, 34.5%
Metformin (*n*, %)	23, 27.4%
Pioglitazone (*n*, %)	5, 6.0%
Insulin (*n*, %)	8, 9.5%
Glucagon-like peptide-1 receptor agonist (*n*, %)	9, 10.7%

**Table 3:** Changes in metabolic parameters after the strart of dotinurad in all patients (*n* = 84).

	*n*	Baseline	After 3 Months	*n*	Baseline	After 6 Months
Body weight (kg)	71	75.7 ± 16.8	75.2 ± 16.3	44	75.7 ± 17.5	75.0 ± 16.8
Systolic blood pressure (mmHg)	75	131.9 ± 18.4	129.1 ± 15.8	45	135.3 ± 20.5	133.9 ± 16.2
Diastolic blood pressure (mmHg)	75	75.8 ± 13.5	74.4 ± 13.0	45	76.0 ± 13.1	76.4 ± 12.8
Plasma glucose (mg/dL)	74	137.6 ± 39.9	135.8 ± 35.3	53	135.8 ± 35.4	134.9 ± 41.9
HbA1c (%)	69	6.6 ± 1.1	6.6 ± 0.9	50	6.5 ± 0.9	6.5 ± 1.0
AST (IU/L)	78	25.3 ± 9.7	26.9 ± 16.0	56	25.5 ± 10.6	28.8 ± 19.0
ALT (IU/L)	76	27.3 ± 19.4	26.3 ± 17.7	55	26.8 ± 19.7	28.1 ± 27.5
TG (mg/dL)	78	176.0 ± 122.8	170.6 ± 97.2	55	156.1 ± 85.0	153.6 ± 83.2
HDL-C (mg/dL)	77	53.4 ± 15.4	53.7 ± 16.3	54	54.0 ± 16.1	55.3 ± 16.6
LDL-C (mg/dL)	68	100.7 ± 26.0	100.1 ± 25.7	50	98.5 ± 25.9	100.1 ± 28.4
Non-HDL-C (mg/dL)	68	130.1 ± 29.0	128.8 ± 28.7	47	127.2 ± 28.8	124.1 ± 29.3
UA (mg/dL)	77	6.6 ± 1.6	6.4 ± 1.4	53	6.7 ± 1.5	6.1 ± 1.2 **
Seum creatinine (mg/dL)	77	1.00 ± 0.39	1.02 ± 0.48	56	1.00 ± 0.33	1.00 ± 0.31
eGFR (ml/min/1.73m^2^)	77	60.0 ± 20.3	59.4 ± 21.1	55	59.9 ± 19.6	59.6 ± 19.5
UACR	42	266.4 ± 458.1	336.6 ± 709.3	26	265.4 ± 400.1	152.2 ± 238.5 *

Values show mean ± SD. * *p* < 0.1, ** *p* < 0.05 vs. baseline. ALT, alanine aminotransferase; AST, aspartate aminotransferase; eGFR, estimated glomerular filtration rate; HDL-C, high-density lipoprotein-cholesterol; LDL-C, low-density lipoprotein-cholesterol; Non-HDL-C, non-high-density lipoprotein-cholesterol; TG, triglyceride, UA, uric acid; UACR, urinary albumin creatinine ratio.

**Table 4:** Changes in metabolic parameters after the start of dotinurad in patients who had not used UA-lowering drugs (*n* = 35).

	*n*	Baseline	After 3 Months	*n*	Baseline	After 6 Months
Body weight (kg)	28	71.8 ± 18.2	71.1 ± 17.3	16	73.2 ± 20.0	73.0 ± 18.5
Systolic blood pressure (mmHg)	31	129.1 ± 19.0	129.5 ± 19.1	16	133.0 ± 20.7	133.2 ± 17.5
Diastolic blood pressure (mmHg)	31	74.0 ± 13.0	73.6 ± 13.1	16	74.4 ± 12.3	76.1 ± 14.6
Plasma glucose (mg/dL)	31	139.2 ± 37.8	137.8 ± 34.3	21	136.2 ± 37.8	134.2 ± 34.3
HbA1c (%)	30	6.8 ± 1.0	6.6 ± 0.6	21	6.7 ± 1.0	6.5 ± 0.8
AST (IU/L)	32	23.6 ± 8.0	24.3 ± 7.3	21	23.2 ± 9.1	24.3 ± 8.5
ALT (IU/L)	32	23.6 ± 13.6	23.2 ± 12.0	21	21.6 ± 9.9	21.0 ± 8.8
TG (mg/dL)	32	159.4 ± 114.3	154.7 ± 101.1	20	135.3 ± 58.3	148.9 ± 83.1
HDL-C (mg/dL)	32	50.8 ± 12.9	53.3 ± 15.8	20	50.5 ± 13.3	50.9 ± 14.5
LDL-C (mg/dL)	27	101.9 ± 29.0	93.9 ± 24.3 **	18	100.3 ± 25.8	98.4 ± 28.0
Non-HDL-C (mg/dL)	26	128.7 ± 32.7	121.2 ± 30.3 *	16	127.2 ± 33.8	123.9 ± 35.5
UA (mg/dL)	32	7.8 ± 1.3	6.3 ± 1.4 **	19	8.0 ± 1.0	6.1 ± 1.3 **
Seum creatinine (mg/dL)	32	0.94 ± 0.19	0.94 ± 0.18	21	0.96 ± 0.20	0.97 ± 0.19
eGFR (ml/min/1.73m^2^)	32	56.9 ± 16.2	56.2 ± 14.8	21	55.1 ± 12.6	55.1 ± 15.3
UACR	20	181.4 ± 230.9	233.4 ± 398.2	13	214.6 ± 398.2	100.4 ± 118.8 *

Values show mean ± SD. * *p* < 0.1, ** *p* < 0.05 vs. baseline. ALT, alanine aminotransferase; AST, aspartate aminotransferase; eGFR, estimated glomerular filtration rate; HDL-C, high-density lipoprotein-cholesterol; LDL-C, low-density lipoprotein-cholesterol; Non-HDL-C, non-high-density lipoprotein-cholesterol; TG, triglyceride, UA, uric acid; UACR, urinary albumin creatinine ratio.

**Table 5:** Changes in metabolic parameters after the strart of dotinurad in patients who switched from xanthine oxidase inhibitors (*n* = 49).

	*n*	Baseline	After 3 Months	*n*	Baseline	After 6 Months
Body weight (kg)	43	78.2 ± 15.5	78.0 ± 15.2	28	77.1 ± 16.2	76.2 ± 15.9 *
Systolic blood pressure (mmHg)	44	133.9 ± 18.0	128.9 ± 13.3 **	29	136.6 ± 20.6	134.3 ± 15.8
Diastolic blood pressure (mmHg)	44	77.0 ± 14.3	75.0 ± 13.0	29	76.9 ± 14.3	75.0 ± 13.0
Plasma glucose (mg/dL)	43	136.5 ± 41.7	134.3 ± 36.4	32	135.6 ± 40.6	135.3 ± 48.4
HbA1c (%)	39	6.5 ± 1.1	6.5 ± 1.1	29	6.4 ± 1.0	6.4 ± 1.1
AST (IU/L)	46	26.5 ± 10.6	28.7 ± 19.9	35	27.0 ± 11.4	31.5 ± 22.9
ALT (IU/L)	44	30.0 ± 22.5	28.5 ± 20.8	34	30.0 ± 23.4	32.4 ± 33.8
TG (mg/dL)	46	187.5 ± 128.4	181.7 ± 93.9	35	167.9 ± 95.8	156.3 ± 84.4
HDL-C (mg/dL)	45	55.3 ± 16.9	54.0 ± 16.7	34	56.1 ± 17.4	57.9 ± 17.4
LDL-C (mg/dL)	41	99.9 ± 24.1	104.2 ± 26.1	32	97.4 ± 26.3	104.2 ± 26.1
Non-HDL-C (mg/dL)	42	130.9 ± 26.8	133.6 ± 26.9	31	127.3 ± 26.4	124.2 ± 26.1
UA (mg/dL)	45	5.8 ± 1.2	6.4 ± 1.5 **	34	5.9 ± 1.2	6.1 ± 1.1
Seum creatinine (mg/dL)	45	1.04 ± 0.49	1.07 ± 0.61	35	1.02 ± 0.39	1.03 ± 0.36
eGFR (ml/min/1.73m^2^)	45	62.2 ± 22.7	61.6 ± 24.5	34	62.8 ± 22.5	62.3 ± 21.4
UACR	22	343.7 ± 590.2	430.5 ± 905.3	13	316.2 ± 514.3	204.0 ± 314.0

Values show mean ± SD. * *p* < 0.1, ** *p* < 0.05 vs. baseline. ALT, alanine aminotransferase; AST, aspartate aminotransferase; eGFR, estimated glomerular filtration rate; HDL-C, high-density lipoprotein-cholesterol; LDL-C, low-density lipoprotein-cholesterol; Non-HDL-C, non-high-density lipoprotein-cholesterol; TG, triglyceride, UA, uric acid; UACR, urinary albumin creatinine ratio.

**Table 6:** Changes in metabolic parameters after the start of dotinurad in patients who had taken anti-hypertensive drugs (*n* = 50).

	*n*	Baseline	After 3 Months	*n*	Baseline	After 6 Months
Body weight (kg)	46	72.6 ± 17.3	72.4 ± 16.9	29	72.0 ± 17.4	71.9 ± 16.8
Systolic blood pressure (mmHg)	49	134.1 ± 18.2	132.3 ± 13.4	30	135.6 ± 19.1	137.0 ± 17.0
Diastolic blood pressure (mmHg)	49	73.9 ± 14.2	73.2 ± 13.2	30	73.0 ± 12.6	75.3 ± 12.9
Plasma glucose (mg/dL)	48	140.5 ± 46.0	134.1 ± 36.1	33	137.7 ± 40.1	137.3 ± 40.4
HbA1c (%)	46	6.6 ± 1.0	6.5 ± 1.0	33	6.5 ± 0.8	6.6 ± 1.1
TG (mg/dL)	49	159.2 ± 113.0	163.5 ± 93.5	34	133.8 ± 65.9	144.7 ± 79.8
HDL-C (mg/dL)	49	54.2 ± 14.2	54.8 ± 16.3	34	54.1 ± 14.4	56.4 ± 15.8
LDL-C (mg/dL)	43	98.4 ± 24.1	99.1 ± 25.6	31	98.4 ± 26.2	99.5 ± 26.7
Non-HDL-C (mg/dL)	43	126.2 ± 27.4	126.1 ± 29.8	28	122.6 ± 26.4	120.5 ± 28.4
UA (mg/dL)	48	7.0 ± 1.5	6.3 ± 1.4 *	32	6.8 ± 1.3	6.1 ± 1.3 **
Urinary UA/creatinine	33	0.36 ± 0.19	0.46 ± 0.24 **	21	0.41 ± 0.20	0.52 ± 0.27 *
Seum creatinine (mg/dL)	49	1.06 ± 0.47	1.08 ± 0.58	34	1.02 ± 0.32	1.03 ± 0.32
eGFR (ml/min/1.73m^2^)	49	56.3 ± 21.6	56.7 ± 23.5	34	56.6 ± 21.9	55.6 ± 21.3
UACR	29	368.0 ± 520.5	443.0 ± 826.5	19	306.6 ± 443.7	152.7 ± 238.1 *

Values show mean ± SD. * *p* < 0.1, ** *p* < 0.05 vs. baseline. eGFR, estimated glomerular filtration rate; HDL-C, high-density lipoprotein-cholesterol; LDL-C, low-density lipoprotein-cholesterol; Non-HDL-C, non-high-density lipoprotein-cholesterol; TG, triglyceride, UA, uric acid; UACR, urinary albumin creatinine ratio.

**Table 7:** Changes in metabolic parameters after the start of dotinurad in patients who had not taken anti-hypertensive drugs (*n* = 34).

	*n*	Baseline	After 3 Months	*n*	Baseline	After 6 Months
Body weight (kg)	25	81.4 ± 14.3	80.3 ± 14.0 *	15	82.9 ± 16.0	80.9 ± 15.5 *
Systolic blood pressure (mmHg)	26	127.8 ± 18.5	123.1 ± 18.4 *	15	134.7 ± 23.7	127.7 ± 13.0 *
Diastolic blood pressure (mmHg)	26	79.3 ± 11.6	76.8 ± 12.6	15	82.1 ± 12.4	78.7 ± 12.6
Plasma glucose (mg/dL)	26	132.3 ± 25.0	138.7 ± 34.3	20	132.7 ± 26.4	130.9 ± 34.3
HbA1c (%)	23	6.7 ± 1.2	6.6 ± 0.8	17	6.6 ± 1.0	6.3 ± 0.7
TG (mg/dL)	29	204.3 ± 135.2	182.6 ± 103.7 *	21	192.1 ± 100.7	167.9 ± 88.6
HDL-C (mg/dL)	28	52.0 ± 17.6	51.6 ± 16.2	20	53.9 ± 19.1	53.3 ± 18.1
LDL-C (mg/dL)	25	104.5 ± 29.2	101.8 ± 26.4	19	98.6 ± 26.0	100.9 ± 31.7
Non-HDL-C (mg/dL)	25	136.7 ± 31.0	133.6 ± 26.6	19	134.0 ± 31.0	129.5 ± 30.5
UA (mg/dL)	29	6.6 ± 1.7	6.6 ± 1.5	21	6.4 ± 1.7	6.1 ± 1.1
Urinary UA/creatinine	21	0.32 ± 0.14	0.51 ± 0.16 **	15	0.29 ± 0.13	0.49 ± 0.26 **
Seum creatinine (mg/dL)	28	0.88 ± 0.15	0.92 ± 0.20 **	22	0.97 ± 0.35	0.96 ± 0.30
eGFR (ml/min/1.73m^2^)	28	66.3 ± 16.1	64.0 ± 15.1 **	21	65.1 ± 14.0	66.0 ± 14.3
UACR	13	39.9 ± 64.6	99.3 ± 192.3	7	151.0 ± 237.6	151.0 ± 258.7

Values show mean ± SD. * *p* < 0.1, ** *p* < 0.05 vs. baseline. eGFR, estimated glomerular filtration rate; HDL-C, high-density lipoprotein-cholesterol; LDL-C, low-density lipoprotein-cholesterol; Non-HDL-C, non-high-density lipoprotein-cholesterol; TG, triglyceride, UA, uric acid; UACR, urinary albumin creatinine ratio.

**Table 8:** Changes in metabolic parameters after the start of dotinurad in patients who had taken statins (*n* = 39).

	*n*	Baseline	After 3 Months	*n*	Baseline	After 6 Months
Body weight (kg)	37	77.6 ± 15.7	77.3 ± 15.1	24	78.6 ± 17.5	78.0 ± 16.2
Systolic blood pressure (mmHg)	38	134.9 ± 20.2	129.4 ± 17.7 **	24	139.0 ± 22.3	135.0 ± 16.8
Diastolic blood pressure (mmHg)	38	77.5 ± 12.9	73.1 ± 13.3 **	24	77.0 ± 13.3	76.4 ± 11.8
Plasma glucose (mg/dL)	38	136.3 ± 37.5	134.2 ± 30.0	27	137.0 ± 34.5	130.4 ± 35.4
HbA1c (%)	37	6.8 ± 1.0	6.8 ± 0.9	27	6.7 ± 0.9	6.8 ± 1.0
TG (mg/dL)	39	169.8 ± 117.5	151.6 ± 71.8	27	152.7 ± 86.8	134.4 ± 70.4
HDL-C (mg/dL)	39	55.1 ± 13.6	54.5 ± 14.1	27	55.9 ± 14.6	57.0 ± 15.3
LDL-C (mg/dL)	35	90.7 ± 22.3	92.2 ± 23.7	23	86.8 ± 19.8	91.1 ± 23.3
Non-HDL-C (mg/dL)	34	121.1 ± 27.1	117.5 ± 25.0	23	116.1 ± 25.1	113.3 ± 22.1
UA (mg/dL)	38	6.5 ± 1.6	6.3 ± 1.4	26	6.6 ± 1.4	6.1 ± 1.0 *
Urinary UA/creatinine	28	0.34 ± 0.17	0.46 ± 0.24 **	18	0.37 ± 0.19	0.51 ± 0.30 **
Seum creatinine (mg/dL)	39	1.03 ± 0.49	1.10 ± 0.62 *	28	1.03 ± 0.32	1.04 ± 0.62
eGFR (ml/min/1.73m^2^)	39	57.4 ± 18.7	56.6 ± 19.4	28	57.5 ± 18.7	56.8 ± 17.1
UACR	19	287.0 ± 464.2	226.4 ± 560.5	12	342.8 ± 488.8	140.5 ± 145.5 *

Values show mean ± SD. * *p* < 0.1, ** *p* < 0.05 vs. baseline. eGFR, estimated glomerular filtration rate; HDL-C, high-density lipoprotein-cholesterol; LDL-C, low-density lipoprotein-cholesterol; Non-HDL-C, non-high-density lipoprotein-cholesterol; TG, triglyceride, UA, uric acid; UACR, urinary albumin creatinine ratio.

**Table 9:** Changes in metabolic parameters after the start of dotinurad in patients who had not taken anti-hyperlipidemic drugs (*n* = 35).

	*n*	Baseline	After 3 Months	*n*	Baseline	After 6 Months
Body weight (kg)	22	74.9 ± 16.4	73.9 ± 15.5 *	16	73.6 ± 16.2	72.5 ± 15.6
Systolic blood pressure (mmHg)	30	129.6 ± 17.1	127.9 ± 13.4	17	130.3 ± 19.8	129.6 ± 13.0
Diastolic blood pressure (mmHg)	30	75.0 ± 14.3	75.6 ± 11.7	17	75.9 ± 14.1	76.2 ± 11.7
Plasma glucose (mg/dL)	30	136.5 ± 45.3	134.3 ± 39.6	22	133.7 ± 39.6	135.0 ± 49.8
HbA1c (%)	26	6.3 ± 0.8	6.2 ± 0.8	19	6.3 ± 0.9	6.1 ± 0.8 **
TG (mg/dL)	32	171.6 ± 103.3	171.4 ± 92.1	23	157.0 ± 91.0	157.1 ± 91.4
HDL-C (mg/dL)	31	51.1 ± 17.0	52.5 ± 18.1	22	51.5 ± 17.9	54.4 ± 19.2
LDL-C (mg/dL)	28	113.0 ± 26.2	110.7 ± 25.8	22	110.0 ± 27.5	109.1 ± 31.2
Non-HDL-C (mg/dL)	27	141.0 ± 30.0	140.0 ± 27.7	19	138.8 ± 31.5	132.4 ± 34.0
UA (mg/dL)	32	6.8 ± 1.6	6.4 ± 1.4	22	6.7 ± 1.6	6.0 ± 1.1 *
Urinary UA/creatinine	22	0.36 ± 0.17	0.51 ± 0.19 **	16	0.35 ± 0.19	0.50 ± 0.23 **
Seum creatinine (mg/dL)	31	0.95 ± 0.27	0.96 ± 0.31	23	1.00 ± 0.37	0.98 ± 0.31
eGFR (ml/min/1.73m^2^)	31	60.8 ± 18.4	59.5 ± 16.5	22	60.2 ± 17.0	61.7 ± 17.6
UACR	17	99.2 ± 164.4	180.4 ± 318.1	12	130.1 ± 226.7	89.6 ± 198.9

Values show mean ± SD. * *p* < 0.1, ** *p* < 0.05 vs. baseline. eGFR, estimated glomerular filtration rate; HDL-C, high-density lipoprotein-cholesterol; LDL-C, low-density lipoprotein-cholesterol; Non-HDL-C, non-high-density lipoprotein-cholesterol; TG, triglyceride, UA, uric acid; UACR, urinary albumin creatinine ratio.

**Table 10:** Changes in metabolic parameters after the start of dotinurad in patients who had taken SGLT2 inhibitors (*n* = 25).

	*n*	Baseline	After 3 Months	*n*	Baseline	After 6 Months
Body weight (kg)	24	77.0 ± 14.8	76.3 ± 14.4	15	76.8 ± 16.2	75.5 ± 15.2
Systolic blood pressure (mmHg)	25	136.5 ± 23.5	133.8 ± 21.0	15	144.9 ± 25.0	143.2 ± 17.0
Diastolic blood pressure (mmHg)	25	78.7 ± 12.9	77.3 ± 14.8	15	77.3 ± 12.8	82.8 ± 14.8 *
Plasma glucose (mg/dL)	25	153.3 ± 43.9	155.0 ± 36.3	16	145.7 ± 28.2	144.1 ± 33.2
HbA1c (%)	25	7.2 ± 1.3	7.0 ± 0.9	16	6.9 ± 0.8	7.0 ± 1.1
TG (mg/dL)	25	215.5 ± 170.5	195.3 ± 123.2	16	160.9 ± 103.8	154.8 ± 96.7
HDL-C (mg/dL)	25	55.5 ± 13.8	54.8 ± 15.8	16	55.9 ± 14.3	56.6 ± 12.9
LDL-C (mg/dL)	21	91.7 ± 21.6	92.4 ± 23.9	14	87.4 ± 20.4	90.8 ± 20.7
Non-HDL-C (mg/dL)	25	123.2 ± 26.6	123.2 ± 29.5	16	112.0 ± 18.4	116.0 ± 22.8
UA (mg/dL)	24	6.7 ± 1.8	7.0 ± 1.6	14	6.8 ± 1.6	6.6 ± 1.2
Urinary UA/creatinine	13	0.33 ± 0.10	0.53 ± 0.23 **	7	0.33 ± 0.10	0.41 ± 0.12
Seum creatinine (mg/dL)	25	1.09 ± 0.55	1.14 ± 0.72	16	1.03 ± 0.26	1.05 ± 0.24
eGFR (ml/min/1.73m^2^)	25	58.9 ± 23.3	58.8 ± 27.1	16	57.1 ± 21.9	56.1 ± 21.4
UACR	17	450.0 ± 654.6	495.4 ± 992.9	9	475.7 ± 575.8	256.7 ± 312.0

Values show mean ± SD. * *p* < 0.1, ** *p* < 0.05 vs. baseline. eGFR, estimated glomerular filtration rate; HDL-C, high-density lipoprotein-cholesterol; LDL-C, low-density lipoprotein-cholesterol; Non-HDL-C, non-high-density lipoprotein-cholesterol; SGLT2, sodium-glucose co-transporter 2; TG, triglyceride, UA, uric acid; UACR, urinary albumin creatinine ratio.

**Table 11:** Changes in metabolic parameters after the start of dotinurad in patients who had not taken SGLT2 inhibitors (*n* = 59).

	*n*	Baseline	After 3 Months	*n*	Baseline	After 6 Months
Body weight (kg)	47	75.0 ± 17.8	74.7 ± 17.3	29	75.1 ± 17.8	74.7 ± 17.8
Systolic blood pressure (mmHg)	50	129.6 ± 15.0	126.8 ± 12.1	30	130.5 ± 16.2	129.3 ± 13.9
Diastolic blood pressure (mmHg)	50	74.3 ± 13.7	73.0 ± 11.9	30	75.4 ± 13.4	73.2 ± 10.5
Plasma glucose (mg/dL)	49	129.6 ± 35.5	126.8 ± 12.1	37	131.5 ± 37.6	131.0 ± 45.0
HbA1c (%)	44	6.3 ± 0.8	6.3 ± 0.8	34	6.4 ± 0.9	6.2 ± 0.8 **
TG (mg/dL)	53	157.3 ± 88.5	158.9 ± 80.9	39	154.1 ± 77.5	153.1 ± 78.4
HDL-C (mg/dL)	52	52.4 ± 16.2	53.1 ± 16.6	38	53.2 ± 16.9	54.7 ± 18.1
LDL-C (mg/dL)	47	104.7 ± 27.0	103.5 ± 26.0	36	102.8 ± 26.7	103.7 ± 30.4
Non-HDL-C (mg/dL)	43	134.1 ± 29.9	132.1 ± 28.0	31	135.1 ± 30.2	128.3 ± 31.6
UA (mg/dL)	53	6.6 ± 1.5	6.1 ± 1.3 **	39	6.6 ± 1.5	5.9 ± 1.2 **
Urinary UA/creatinine	41	0.35 ± 0.18	0.47 ± 0.21 **	29	0.37 ± 0.20	0.53 ± 0.29 **
Seum creatinine (mg/dL)	52	0.95 ± 0.28	0.96 ± 0.30	40	0.99 ± 0.36	0.99 ± 0.33
eGFR (ml/min/1.73m^2^)	52	60.5 ± 18.9	59.6 ± 17.8	39	61.0 ± 18.9	61.0 ± 18.7
UACR	25	141.6 ± 180.8	228.6 ± 415.1	17	154.1 ± 215.3	96.9 ± 175.5

Values show mean ± SD. ** *p* < 0.05 vs. baseline. eGFR, estimated glomerular filtration rate; HDL-C, high-density lipoprotein-cholesterol; LDL-C, low-density lipoprotein-cholesterol; Non-HDL-C, non-high-density lipoprotein-cholesterol; SGLT2, sodium-glucose co-transporter 2; TG, triglyceride, UA, uric acid; UACR, urinary albumin creatinine ratio.

**Table 12:** Changes in metabolic parameters after the start of dotinurad in patients who had not taken anti-hypertensive and anti-hyperlipidemic drugs and SGLT2 inhibitors (*n* = 13).

	*n*	Baseline	After 3 Months	*n*	Baseline	After 6 Months
Body weight (kg)	10	80.2 ± 8.2	78.4 ± 8.4 *	6	76.8 ± 12.6	75.4 ± 13.0
Systolic blood pressure (mmHg)	11	123.7 ± 15.3	121.8 ± 12.3	6	130.8 ± 25.7	127.0 ± 12.1
Diastolic blood pressure (mmHg)	11	76.2 ± 7.5	77.4 ± 8.0	6	78.8 ± 9.7	78.5 ± 10.3
Plasma glucose (mg/dL)	11	126.4 ± 24.7	126.0 ± 34.1	8	127.4 ± 25.9	129.4 ± 54.2
HbA1c (%)	8	6.3 ± 0.9	6.4 ± 0.9	5	6.4 ± 1.2	5.9 ± 0.8 *
TG (mg/dL)	13	187.0 ± 110.7	172.7 ± 107.2	9	180.8 ± 120.4	150.9 ± 96.9
HDL-C (mg/dL)	12	49.3 ± 22.1	51.1 ± 19.9	8	50.4 ± 25.4	52.1 ± 25.0
LDL-C (mg/dL)	11	111.2 ± 31.2	112.5 ± 25.6	8	107.1 ± 35.7	109.5 ± 39.6
Non-HDL-C (mg/dL)	11	143.1 ± 33.1	146.4 ± 22.8	9	143.6 ± 37.0	139.2 ± 35.0
UA (mg/dL)	13	6.7 ± 1.9	6.2 ± 1.4	9	6.5 ± 2.1	6.0 ± 1.3
Urinary UA/creatinine	10	0.36 ± 0.18	0.56 ± 0.15 **	6	0.33 ± 0.19	0.65 ± 0.29 **
Seum creatinine (mg/dL)	12	0.82 ± 0.12	0.85 ± 0.14	9	1.00 ± 0.52	0.96 ± 0.44
eGFR (ml/min/1.73m^2^)	12	70.2 ± 14.3	67.4 ± 13.8	8	68.5 ± 12.0	73.0 ± 15.7 *
UACR	5	24.3 ± 17.1	151.2 ± 275.1	4	185.3 ± 316.9	192.3 ± 348.7

Values show mean ± SD. * *p* < 0.1, ** *p* < 0.05 vs. baseline. eGFR, estimated glomerular filtration rate; HDL-C, high-density lipoprotein-cholesterol; LDL-C, low-density lipoprotein-cholesterol; Non-HDL-C, non-high-density lipoprotein-cholesterol; SGLT2, sodium-glucose co-transporter 2; TG, triglyceride, UA, uric acid; UACR, urinary albumin creatinine ratio.

## Data Availability

The data supporting the findings of this study are available from the corresponding author upon reasonable request.
